# Microwave Polarization Sensing for Dielectric Materials Based on a Twisted Dual-Layer Meta-Surface

**DOI:** 10.3390/ma15196655

**Published:** 2022-09-26

**Authors:** Hong Xiao, Sen Yan, Juan Chen

**Affiliations:** School of Information and Communications Engineering, Xi’an Jiaotong University, Xi’an 710049, China

**Keywords:** microwave sensor, meta-surface, polarization sensing, dielectric materials, polarization conversion

## Abstract

A microwave sensor is proposed based on a chiral twisted dual-layer meta-surface. Elliptical angle and polarization rotation angle are used to characterize the different dielectric constants of materials. The dielectric films consisting of polydimethylsiloxane and barium titanate with volume fractions 0%, 10%, 15%, 20% are prepared and tested for a proof of concept. The measured results show that the Q factors of polarization rotation angle and elliptical angle peak are 11.85 when the volume fraction of barium titanate is 20%, which is 75.5% higher than 6.75 of the transmission resonance peak, and the figures of merit of the polarization rotation angle and elliptical angle peak are 0.99 and 0.86, which are 73.7% and 50.9% higher than the 0.57 of transmission resonance, respectively. Compared to the resonance sensing method, polarization sensing not only has a better Q factor and figure of merit while maintaining similar sensitivity, but also obtains more characterization information due to the double-parameter sensing, which provide a new idea for the development of high-sensitivity microwave sensors.

## 1. Introduction

Meta-surfaces (MSs) are quasi-2-D artificial structures formed from subwavelength periodic arrays of unit cells, which can manipulate the amplitude, phase, and polarization states of electromagnetic (EM) waves. With the characteristic of local electric field enhancement, MSs are extremely sensitive to variations in the surrounding dielectric environment and, thus, have been also explored for various sensing applications [1,2,3,4,5]. Indeed, H. K. Kim et al. introduced a MS absorber for detecting the ethanol concentration from the resonance frequency shift of the absorber [1]. Additionally, Z. J. Cui et al. demonstrated an all-silicon plasmon MS to be a highly sensitive biosensor for the Bacillus thuringiensis Cry1Ac toxin [2]. Furthermore, W. Su et al. proposed a graphene-coated all-dielectric MS with multi-fano resonances for refractive index sensing [3]. For MS sensors, detecting the physical parameter changes in samples is mainly based on the resonance sensing method that relies on monitoring the frequency shifts of the resonance peak. In most designs, the changes in their polarization state are generally ignored.

When MS has a dual-layer or multi-layer twisted coupling structure, it has intrinsic chirality [6,7,8]. Moreover, a single-layer anisotropic metallic structure can also exhibit extrinsic chirality when the EM wave incidents obliquely [9,10]. Chiral MSs can break the mirror symmetry and realize polarization conversion and asymmetric one-way transmission [11,12]. In recent years, chiral MSs have been reported to be used for THz polarization sensing and identification for biomaterials [10,11,13]. For example, in [10], a single-layer chiral MS was proposed for quantitative concentration analysis of three amino acid solutions and qualitative discrimination between their D- and L- enantiomers. Four polarization parameter spectra, e.g., elliptical angle (EA), polarization rotation angle (PRA), circular dichroism (CD), and optical activity (OA) spectra were used for sensing characterization. In [13], a dual-layer twisted MS was used as a sensor to realize the thermal denaturation sensing, concentration sensing, and type identification of protein aqueous solutions. The right-hand circular polarization (RCP) spectra were used for polarization sensing characterization. The polarization sensing method depends on tracking the changes in polarization state, which can obtain more effective information about the characteristics of the biological samples than resonance sensing. However, the works related to microwave polarization sensing by using chiral MS have not been reported yet.

The dielectric constant is a measure of the dielectric material response when an external electric field is applied to it [14]. Microwave dielectric measurement of materials is very important in various areas, such as the food industry, agriculture, and medicine [15]. In this paper, a twisted dual-layer chiral meta-surface is proposed for dielectric sensing application at microwave frequencies. Two polarization parameter spectra, e.g., the elliptical angle and polarization rotation angle, are used to characterize the different dielectric constants of materials. The unit cell structure of the meta-surface is firstly modeled and simulated, then the meta-surface is fabricated and tested by several dielectric films with different permittivities. The polarization sensing properties were experimentally investigated and finally compared with resonance sensing, and they show better sensing performance.

## 2. Structure Design and Analysis

### 2.1. The Unit Cell of Twisted Dual-Layer Metasurface 

**Figure 1:**
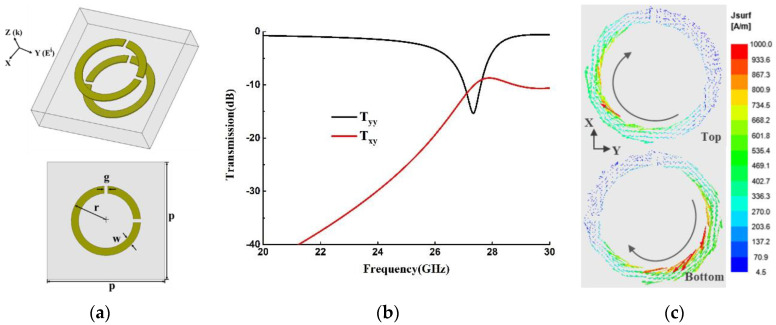
(**a**) Unit cell structure and its geometric parameters are as follows: p = 3.2 mm, r = 0.95 mm, w = 0.2 mm, and g = 0.1 mm. (**b**) Transmission coefficients of the transmitted wave with y-polarized incidence. (**c**) Surface current flow on the top and bottom layer of the meta-surface at 27.3 GHz.

The proposed meta-surface working in the K-band is composed of two layers of metallic structures and one layer of F4b substrate sandwiched in the middle. Shown in Figure 1a, each metallic layer is based on an asymmetric double-opening (ADO) ring structure, and the bottom layer is rotated $90^{\circ }$ with respect to the top one along the z-axis. The structural parameters are also defined in Figure 1a. The structure is modeled and simulated by Ansoft HFSS. The periodic boundary conditions are set in both the x and y directions of the unit cell, and the Floquet ports are used for excitations. Assuming that the incident wave is a y-polarized wave propagating along the z-axis, the transmission coefficients of its transmitted wave are shown in Figure 1b. There is a 16-dB resonant dip excited at 27.3 GHz on the co-polarization transmission curve, which is induced by the magnetic dipole resonance, as shown in Figure 1c. 

**Figure 2:**
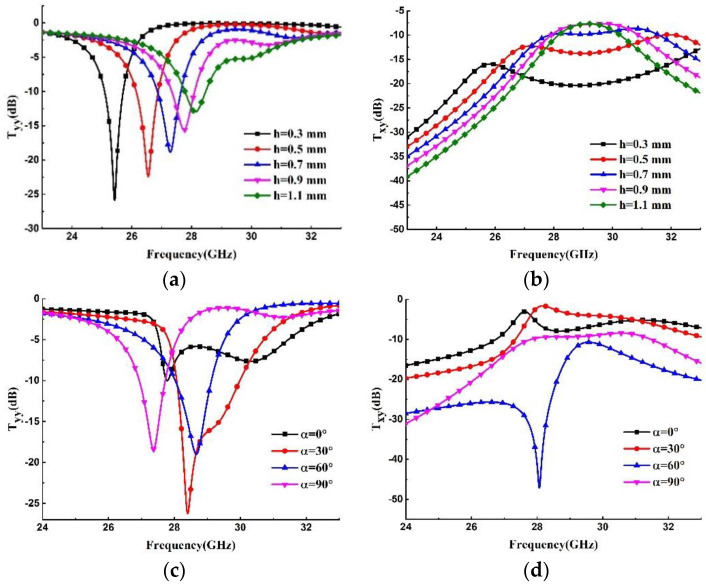
(**a**) Simulated co- and (**b**) cross-polarization transmission curves varying with the thickness h. (**c**) Simulated co- and (**d**) cross-polarization transmission curves varying with the twisted angle α.

The resonant dip is selected to be used for resonance sensing, and the thickness h of the substrate and the twisted angle α between two metallic layers are the key parameters in the simulations, for achieving a sharp resonance peak. Figure 2a shows the simulated co-polarization transmission curves varying with the thickness h. With the decrease in h, the resonant dip moves to lower frequencies, and sharper and sharper resonance peaks can be obtained due to the stronger coupling between the magnetic dipole modes of the two ADO rings. However, if the thickness is less than 0.5 mm, the substrate becomes flexible and is not convenient for testing, while if the thickness exceeds 1.1 mm, the coupling between two metallic layers is too small to form a sharp resonance dip. Here, the available thickness 0.73 mm is selected from standard substrates. Figure 2c shows the simulated co- polarization transmission curves varying with the twisted angle α. With the increase in α, the magnetic dipole modes of two ADO rings gradually approach and, thus, resonate at the same frequency after exceeding 30°. From 30° to 90°, the resonant dip moves to lower frequencies. Here, a conventional twisted angle of 90° is selected. Figure 2b,d show the simulated cross-polarization transmission curves varying with the thickness h and the twisted angle α, respectively.

### 2.2. Polarization State

For a y-polarized wave vertically incident on the meta-surface, the polarization state of the transmitted wave can be defined by elliptical angle (EA), χ and polarization rotation angle (PRA), ψ, which can be calculated as follows [16]:

(1) $$\chi =\frac{1}{2}\arcsin\left(\frac{2q_{t}\sin(\Delta \varphi_{yx})}{1+{\left|q_{t}\right|}^{2}}\right)$$

(2) $$\psi =\frac{1}{2}\arctan\left(\frac{2q_{t}\cos(\Delta \varphi_{yx})}{1-{\left|q_{t}\right|}^{2}}\right)$$

where $q_{t}=\left|t_{xy}\right|/\left|t_{yy}\right|$ is the magnitude ratio of the co- and cross-polarization transmission coefficients, and $\Delta \varphi_{yx}=\varphi_{yy}-\varphi_{xy}$, is the phase difference of them. Elliptical angle χ reflects the polarization state of transmitted wave, and ranges from $-45^{\circ }$ to $+45^{\circ }$, where $0^{\circ }$ denotes a linearly polarized (LP) wave, and $\pm 45^{\circ }$ denotes a left-handed or right-handed circularly polarized wave. Polarization rotation angle ψ reflects the rotation angle of the polarization direction of the transmitted wave with respect to the incident wave, and ranges from $-90^{\circ }$ to $+90^{\circ }$, where the positive values represent the clockwise direction, and the negative values represents the counterclockwise direction. 

**Figure 3:**
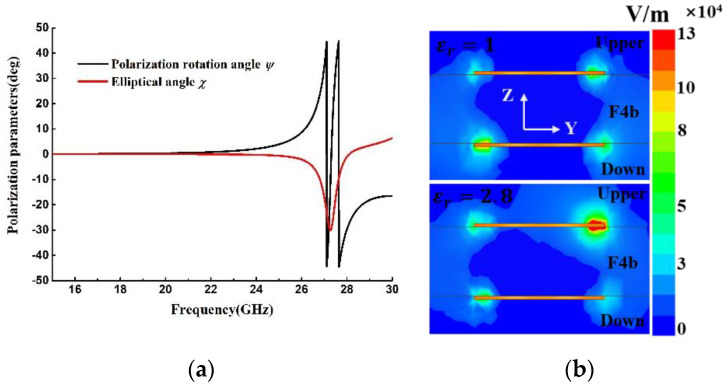
(**a**) Simulated EA and PRA of the transmitted wave with y-polarized incidence. (**b**) Electric field distributions of the meta-surface covered with a sample with the dielectric constant of 2.8 and the thickness of 0.5 mm.

**Figure 4:**
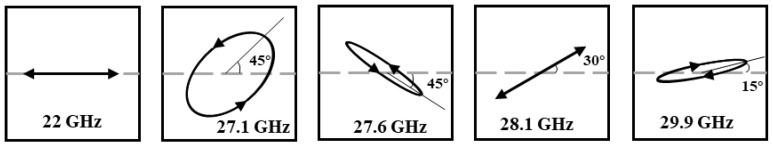
Several polarization states of transmitted wave with y-polarized incidence.

According to Formulas (1) and (2), the simulated EA and PRA of the transmitted wave with y-polarized incident wave are shown in Figure 3a. Several polarization states at different frequencies are plotted in Figure 4. The transmitted wave is y-LP at 22 GHz. After that, the wave becomes right-handed elliptical near the resonant dip at 27.3 GHz, and its major axis rotates with respect to y-axis. The wave continues rotating and stays right-handed elliptical up to 28.1 GHz, where a LP wave is formed with an angle of 30° with respect to the y-axis. After that, the wave gradually becomes a left-handed elliptical.

Moreover, we can see that the EA and PRA have special spectral peaks similar to the transmission resonance peak, which could be used for polarization sensing. Figure 3b shows the electric field distributions of the meta-surface covered with a sample with the dielectric constant of 2.8 and the thickness of 0.5 mm, in the y–z plane at 27.3 GHz. It can be seen that the field is enhanced locally near the metallic structure, and field distribution will be varied due to the variation of the dielectric constant of the sample, leading to the change in the intensity and polarization state of the transmitted wave.

## 3. Experimental Methods and Results 

### 3.1. Measurement Method

**Figure 5:**
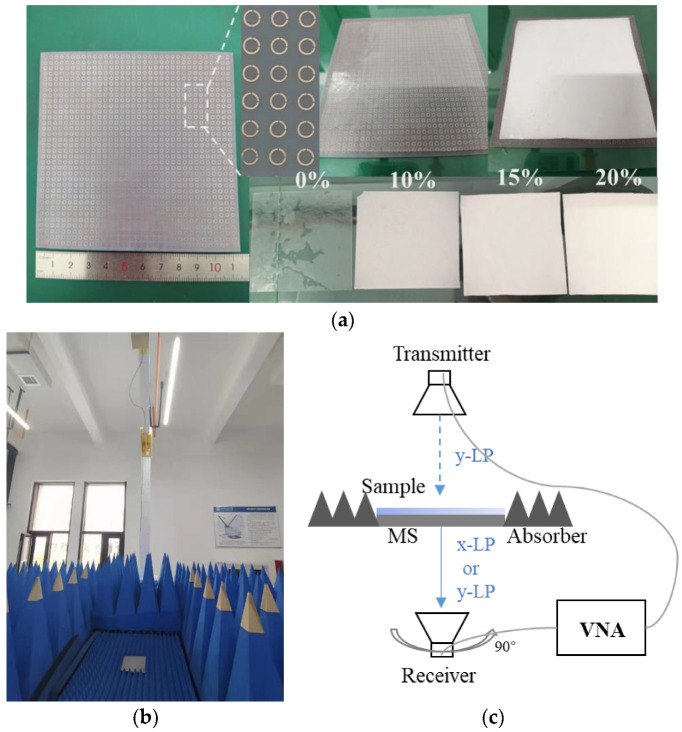
(**a**) Meta-surface prototype and dielectric films made of PDMS and BTO with volume fractions of 0%, 10%, 15%, and 20%. (**b**) The measurement setup and (**c**) its schematic illustration.

The MS prototype was fabricated using standard print circuit board (PCB) technology, which is composed of 34 × 34 unit cells with the total size of 10.9 cm × 10.9 cm, and its photo is given in Figure 5a. The measurements were carried out by the free space method, and the measurement setup and its schematic illustration are shown in Figure 5b,c, respectively. A pair of standard gain horn antennas working in the K-band was placed on both sides of the MS, and the antennas were connected to a vector network analyzer. The incident was chosen as y-LP wave, and the amplitudes and phases of co- and cross-polarization transmission coefficients were measured based on two horns placed parallel and orthogonal, respectively. In the sensing experiments, four film samples were adhered to the upper surface of MS, and each film was measured three times under the same conditions to reduce experimental errors.

### 3.2. Dielectric Matetials Preparation

We chose dielectric films consisting of different proportions of polydimethylsiloxane (PDMS) ($\varepsilon_{r}∼2.8$, tanδ∼0.04) and barium titanate (BTO) ($\varepsilon_{r}∼500$, tanδ∼0.5) for experimental testing. The BTO powders were uniformly mixed in liquid PDMS with a mixer, then poured into the mold, and finally placed on the heating plate and heated at 90° for 30 min, to form 1 mm thick dielectric films. Figure 5a shows the appearance of films made of PDMS and BTO with different volume fractions. The thin films exhibit good adhesion, attributed to the physical characteristics of PDMS, which can greatly reduce the air gap between the films and the meta-surface, leading to small errors in the testing. The dielectric properties of films are estimated using the following Maxwell–Garnett (MG) mixing rule [17,18], as follows:

(3) $$\varepsilon_{d}\left(v\right)=\varepsilon_{1}+3v\varepsilon_{1}\frac{\varepsilon_{2}-\varepsilon_{1}}{\varepsilon_{2}+2\varepsilon_{1}-v\left(\varepsilon_{2}-\varepsilon_{1}\right)}$$

where $\varepsilon_{1}$, $\varepsilon_{2}$, and $\varepsilon_{d}$ are the complex permittivity of PDMS, BTO and the films, respectively, and *v* is the volume fraction of BTO in PDMS. The theoretical dielectric constants of the films are 2.8, 3.6, 4.2, and 4.7, corresponding to BTO volume fractions 0%, 10%, 15%, 20%, respectively. 

### 3.3. Polarization Sensing

**Figure 6:**
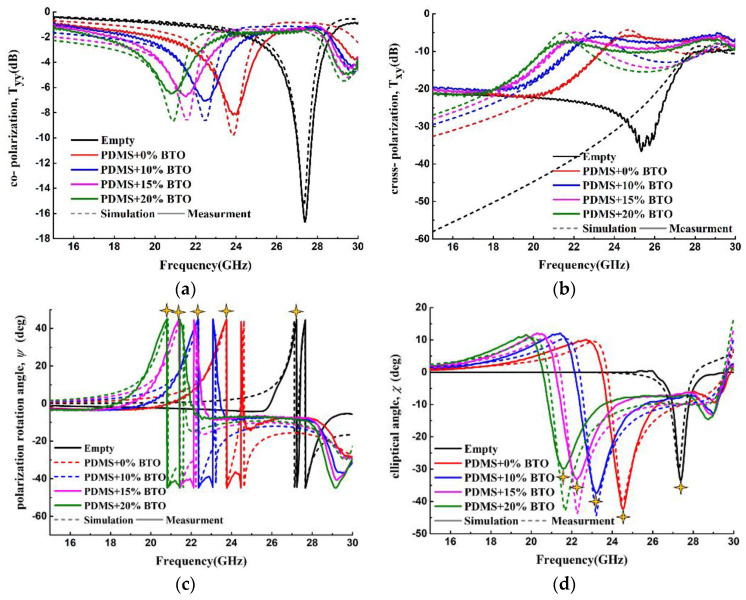
Simulated and measured curves of the (**a**) co- and (**b**) cross-polarization transmission coefficients, and the (**c**) PRA and (**d**) EA, varying with the volume fraction of BTO, respectively.

Finally, we discuss the polarization sensing performance of the meta-surface compared with its resonance sensing. Figure 6a–d show the simulated and measured curves of the co- and cross-polarization transmission coefficients, and the PRA and EA, varying with different dielectric films, respectively. It can be seen that all peak frequencies decreased with the increase in volume fraction of BTO, and the measurement results have good consistency with the simulations. To have more accurate results, in our simulations, an air gap of 2 μm between the film and the sensor is considered. The co-polarization transmission resonance (TR) peak shown in Figure 6a is used as the resonance sensing parameter, and the EA and PRA peak, marked by stars in Figure 6c,d are used as the polarization sensing parameters. 

The figure of merit (*FOM*) is usually used to comprehensively evaluate the sensing performance of the devices. The *FOM* is calculated as follows:

(4) $$FOM=S\times Q/f_{0}=\Delta f/(FWHM\times \Delta \varepsilon_{r})$$

(5) $$S=\Delta f/\Delta \varepsilon_{r},\;Q=f_{0}/FWHM$$

where *S* is the sensitivity, *Q* is the quality factor, *f*_0_ is the peak frequency, and Δf is the peak frequency shift. The *FWHM* is the full width at half maximum, and $\Delta \varepsilon_{r}$ is the dielectric constant change in the films.

**Figure 7:**
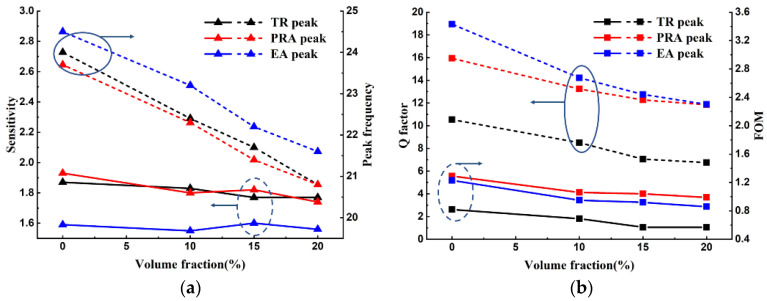
(**a**) Measured frequency shifts and sensitivity, and (**b**) Q factor and FOMs of TR, EA, and PRA peaks, varying with the volume fraction of BTO.

Figure 7a shows the measured peak frequency shifts of TR, EA, and PRA with the changes in volume fraction of BTO. The sensitivities of these three sensing parameters are also shown in Figure 7a. Figure 7b shows the *Q* factors and FOM of the TR, EA, and PRA peaks, varying with the volume fraction of BTO. It can be seen from Figure 7a that the variation trend and range of peak frequency for three sensing parameters are nearly the same. The sensitivities of TR and PRA peak are nearly close, and larger than that of the EA peak. When the volume fraction is 20%, the sensitivities of TR and PRA are 1.77 and 1.74, which are 11.5% and 13.4% higher than the 1.56 of EA, respectively. The relatively steady curves indicate that the sensitivity of the sensor is quite stable. The Q factors of the PRA and EA peaks are much larger than that of the TR peak, as shown in Figure 7b. With the increase in volume fraction of BTO, the Q factors of them decrease gradually, and when the volume fraction is 20%, the Q factors of PRA and EA are nearly same, which is 11.85 and 75.5% higher than the 6.75 of TR. Consequently, the FOMs of the PRA and EA peaks are larger than that of TR peak. When the volume fraction is 20%, the FOMs of PRA and EA are 0.99 and 0.86, which is 73.7% and 50.9% higher than the 0.57 of TR, respectively. Comparatively, the PRA peak has a superior sensing performance. The EA peak has a lower sensitivity than the TR peak, but it has a much higher Q factor and, thus, has a better sensing performance. In other words, compared to the resonance sensing method, the polarization sensing method has a robust performance (higher Q factor) which is less affected by the testing system and the type of samples, while maintaining similar sensitivity. In addition, two polarization sensing parameters, the EA and PRA peaks, bring more characterization information for dielectric materials under test.

## 4. Conclusions

In conclusion, a twisted dual-layer meta-surface is proposed in the K-band. It can be used as a microwave sensor to detect the changes in the dielectric constant of films made of PDMS and BTO with volume fractions of 0%, 10%, 15%, and 20%. Using the same meta-surface device, the polarization sensing method will have better sensing performance than the resonance sensing method, which provides a new physical perspective for the development of highly sensitive sensors. To improve the sensitivity and Q factor of the sensor, the meta-surface with a structural design of a stronger local electric field would be considered. Meanwhile, more dielectric films with different volume fractions of BTO should be measured to reduce measurement error. In the future, liquid sample characterization combined with microfluidic channel could be conducted, and the flexible substrate could also be analyzed and used.
